# Dose-dependent reduction of optic nerve sheath diameter during graded lower body negative pressure in healthy adults of both sexes

**DOI:** 10.3389/fnetp.2026.1908460

**Published:** 2026-08-07

**Authors:** Andrej Bergauer, Janez Urevc, Bianca Steuber, Karin Schmid-Zalaudek, Vid Pivec, Nandu Goswami

**Affiliations:** 1 Division of Physiology, Otto Loewi Research Center of Vascular Biology, Immunity and Inflammation, Medical University of Graz, Graz, Austria; 2 Department of Surgery, General Hospital (LKH Südweststeiermark), Wagna, Austria; 3 Faculty of Mechanical Engineering, University of Ljubljana, Ljubljana, Slovenia; 4 Center for Space and Aviation Health, Mohammed Bin Rashid University of Medicine and Health Sciences, Dubai, United Arab Emirates

**Keywords:** cardiovascular physiology, central hypovolemia, intracranial pressure, lower body negative pressure, network physiology, optic nerve sheath diameter, sex differences, ultrasound

## Abstract

**Background:**

Optic nerve sheath diameter (ONSD) measured by ultrasound provides a non-invasive surrogate of intracranial pressure. Although lower body negative pressure (LBNP) is widely used to simulate central hypovolemia, the dose-response relationship between graded LBNP and ONSD, and possible sex differences therein, are incompletely characterized.

**Methods:**

Forty-four healthy young adults (22 female, 22 male) underwent graded LBNP ( −10 to −40 mmHg) in the supine position; valid ONSD data were available in 37 participants (18 female, 19 male) who constituted the analytical sample. ONSD was measured from transbulbar B-mode ultrasound images 3 mm posterior to the globe, with three representative frames averaged per condition. Relative changes from individual supine baseline were analyzed using a linear mixed-effects model with state, sex, and their interaction as fixed effects.

**Results:**

A progressive and statistically significant reduction in ONSD was observed at every LBNP level in both sexes. Female participants showed reductions of approximately −4.4% (LBNP10, p = 0.001), −9.1% (LBNP20, p < 0.001), −13.2% (LBNP30, p < 0.001) and −15.9% (LBNP40, p < 0.001) relative to supine baseline. Male-specific contrasts showed comparable reductions (LBNP10: −3.0% , p = 0.022; LBNP20: −7.9% , p < 0.001; LBNP30: −10.1% , p < 0.001; LBNP40: −13.4% , p < 0.001). The main effect of sex was not significant (p = 1.00), and no state × sex interaction reached significance (all p > 0.1). ONSD returned to baseline during recovery (female: p = 0.173; male: p = 0.059).

**Conclusion:**

Graded LBNP induces a progressive, dose-dependent reduction in ONSD that is detectable already at mild LBNP ( −10 mmHg) and reaches approximately −15% at −40 mmHg. No statistically significant sex-related differences in the ONSD response to graded LBNP were detected in the present study. These findings support the use of transbulbar ONSD ultrasound as a sensitive non-invasive marker consistent with intracranial-pressure reductions during simulated central hypovolemia. By linking a systemic cardiovascular perturbation to a cerebro-ocular pressure readout, the study offers a network-physiology view of central-hypovolemia responses relevant to spaceflight and orthostatic-stress research.

## 1 Introduction

**FIGURE 1:**
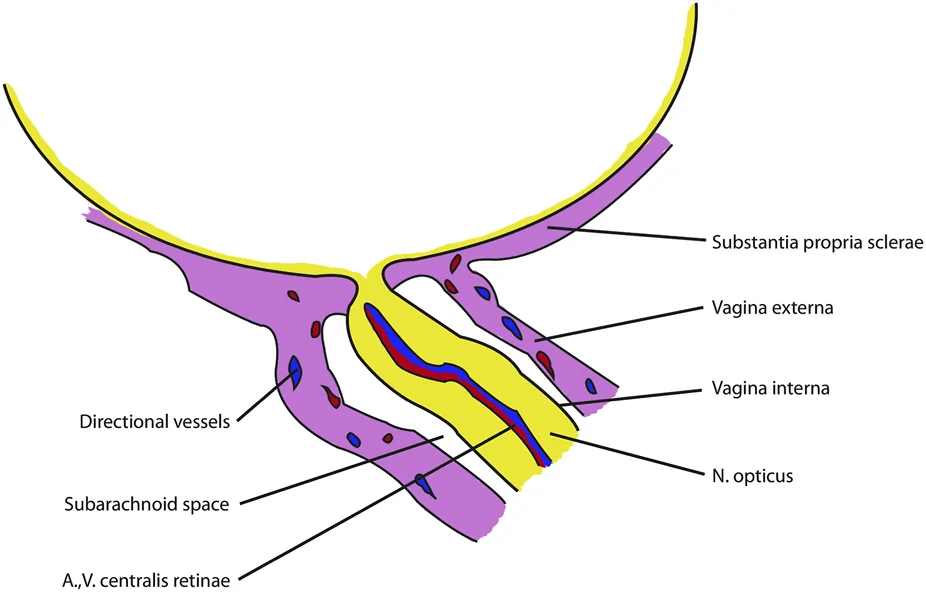
Anatomy of the optic nerve sheath complex in transverse cross-section. The optic nerve (n. opticus) is enclosed by the dural vagina externa with the inner vagina interna; between them lies the subarachnoid space, which is continuous with the intracranial cerebrospinal fluid compartment. Elevations in intracranial pressure therefore distend the vagina externa and increase the optic nerve sheath diameter as measured by transbulbar ultrasound. The a. and v. centralis retinae run axially within the nerve; directional vessels in the tunica externa mark the outer border of the subarachnoid space. Structures are not drawn to scale. Figure reproduced from Bergauer et al. (2017) under the Creative Commons Attribution 4.0 International License (CC BY 4.0).

Intracranial pressure (ICP) is tightly homeostatically regulated and is modulated by posture, central blood volume, and cerebral venous outflow. Direct measurement of ICP requires invasive access and is therefore unsuitable for physiological studies in healthy volunteers. Transbulbar ultrasonography of the optic nerve sheath diameter (ONSD) has emerged as a non-invasive, reproducible surrogate of ICP, exploiting the continuity between the intracranial subarachnoid space and the perioptic subarachnoid space surrounding the retrobulbar optic nerve, which is enclosed by the dural vagina externa (Figure 1) (Helmke and Hansen, 1996; Soldatos et al., 2009). Systematic reviews and meta-analyses have confirmed pooled sensitivity of ∼ 0.90 and specificity of ∼ 0.85 for ONSD-based detection of intracranial hypertension, with a consistent threshold of approximately 5.7–5.9 mm associated with ICP > 20 mmHg (Dubourg et al., 2011; Robba et al., 2018; Montorfano et al., 2021). Validation against direct intraparenchymal ICP monitoring, magnetic-resonance measurements of the sheath, and diverse clinical contexts has established ONSD ultrasound as a robust, modality-independent index of changes in intracranial cerebrospinal fluid (CSF) pressure (Geeraerts et al., 2007; Soldatos et al., 2008; Geeraerts et al., 2008; Liu et al., 2017). The sheath responds within seconds to acute CSF drainage, and because only the sheath, not the optic nerve, distends, ONSD reflects perioptic CSF pressure (Moretti et al., 2009; Geeraerts et al., 2008).

Anatomically, the retrobulbar optic nerve is enveloped by a meningeal sheath continuous with the intracranial dura mater, containing CSF in direct communication with the basal cisterns (Helmke and Hansen, 1996). The most distensible segment lies ∼ 3 mm posterior to the globe, where transmural CSF pressure changes produce the largest proportional change in sheath caliber (Newman et al., 2002; Helmke and Hansen, 1996; Steinborn et al., 2011); this underpins the now-standard measurement protocol adopted in essentially every subsequent validation study (Blaivas et al., 2003; Geeraerts et al., 2007; Tayal et al., 2007; Soldatos et al., 2008; 2009; Rajajee et al., 2011; Dubourg et al., 2011; Robba et al., 2018), for which standardized acquisition protocols have since been proposed to reduce measurement variability (Aspide et al., 2020). In trained hands, reproducibility is high (intra- and inter-observer standard deviations ≈ 0.1–0.3 mm), comparable to the acute ONSD changes seen in physiological challenges such as high-altitude exposure (Karakitsos et al., 2006; Moretti et al., 2009; Sutherland et al., 2008; Fagenholz et al., 2009).

Lower body negative pressure (LBNP) is an established experimental model for inducing graded central hypovolemia by redistributing blood volume toward the lower extremities, reproducing a continuum of central blood-volume losses comparable to controlled hemorrhage while remaining a reversible, well-tolerated stimulus in healthy volunteers (Cooke et al., 2004; Wolthuis et al., 1974; Hinojosa-Laborde et al., 2014; Goswami et al., 2019a; Goswami, 2023). Crucially for the present study, supine LBNP isolates the contribution of reduced central blood volume from the confounding hydrostatic effects that dominate during head-up tilt or standing (Ogoh et al., 2022; Taneja et al., 2007), so that the resulting fall in central venous and thoracic pressures can be attributed to intravascular volume redistribution. Recent invasive work has further shown that lowering central venous pressure (CVP) produces a proportional reduction in directly measured ICP, consistent with the Davson model in which CSF formation is opposed by dural venous-sinus pressure (Hansen et al., 2021; Petersen et al., 2019). Whereas arterial-pressure regulation, autonomic responses, and venous hemodynamics during LBNP are well characterized (Shankhwar et al., 2023; Goswami, 2023), the ONSD response to progressive LBNP-induced central hypovolemia has been examined in only a single previous study, which did not address sex differences (Marshall-Goebel et al., 2017b).

Interest in LBNP and ONSD has been intensified by the observation that prolonged microgravity causes a sustained cephalad fluid shift that elevates ICP, distends the optic nerve sheath, and produces a characteristic constellation of ocular findings, including globe flattening, choroidal folds, hyperopic shifts, and optic disc edema, collectively termed spaceflight-associated neuro-ocular syndrome (SANS) (Mader et al., 2011; Mader et al., 2021; Lee et al., 2020; Martin Paez et al., 2020; Ly et al., 2022). ONSD distention and optic-nerve-head changes have been documented during microgravity (Mader et al., 2021; Marshall-Goebel et al., 2017b), while ground-based head-down-tilt analogs reproduce the increase in ONSD, ICP, and intraocular pressure (Marshall-Goebel et al., 2017b; Marshall-Goebel et al., 2017a; Watkins et al., 2017). In this context, LBNP has been proposed as a mechanical countermeasure that translocates fluid caudad and may acutely reduce both ICP and ONSD; supportive evidence includes direct measurements during head-down tilt in healthy volunteers (Marshall-Goebel et al., 2017b; Watkins et al., 2017; Petersen et al., 2019) and the observation that smaller perivascular-space expansion has been reported in Russian cosmonauts, who routinely use LBNP-based countermeasures, compared with NASA astronauts (Wostyn et al., 2022), although other operational and training factors may also contribute. Whether ONSD responds in a graded, dose-dependent fashion to mild and moderate LBNP in the absence of head-down tilt, and whether the response differs between sexes given known sex differences in orthostatic tolerance (Convertino, 1998; Evans et al., 2018; Goswami et al., 2019a; Goswami et al., 2021; Goswami et al., 2026b; Goswami et al., 2026a), has not been characterized.

More broadly, the interplay between systemic hemodynamics and cerebro-ocular pressure exemplifies the perspective of network physiology, which examines how dynamically interacting organ systems and sub-systems generate integrated physiological states across multiple spatial and temporal scales (Bashan et al., 2012; Ivanov, 2021). Within this framework, graded LBNP perturbs the cardiovascular network (central blood volume, venous return, and autonomic and vascular control), and the resulting change in ONSD provides a non-invasive, multiscale readout of how that perturbation propagates to the craniospinal and ocular compartments. Characterizing this cardiovascular–cerebrovascular–ocular coupling under controlled central hypovolemia is directly relevant to network-physiology approaches to spaceflight and orthostatic stress (Goswami et al., 2026a).

Therefore, the primary aim of this study was to quantify changes in ONSD during graded supine LBNP ( −10 to −40 mmHg) in healthy young adults using transbulbar B-mode ultrasonography. A secondary aim was to determine whether these responses differ between males and females. We hypothesized that graded LBNP would produce a progressive, dose-dependent reduction in ONSD, consistent with a CVP-mediated reduction in ICP.

The present study analyzed ONSD ultrasound measurements collected as part of the experimental protocol and participant cohort previously described by Shankhwar et al. (2023). While that study reported systemic cardiovascular and autonomic responses to graded LBNP, the ONSD data acquired during the same experimental sessions have not been reported previously.

## 2 Materials and Methods

The study was conducted at the Division of Physiology, Medical University of Graz, Austria. Ethical approval was obtained from the institutional ethics committee (Ref. EK 25-551 ex 12/13), and all procedures conformed to the Declaration of Helsinki. All participants received a detailed explanation of the study protocol and provided written informed consent prior to inclusion.

Details of participant recruitment and the experimental protocol have been described previously by Shankhwar et al. (2023). Briefly, young, healthy volunteers aged 18–30 years were recruited. Inclusion criteria comprised non-smoking status and absence of known cardiovascular, thrombotic, neurological, or psychological disorders; female participants were not pregnant at the time of testing and, as documented during screening, none were using hormonal (oral) contraception. Participants were instructed to refrain from endurance exercise for 48 h and from caffeine consumption for 24 h prior to the experiment.

A total of 44 individuals were enrolled in the study. Baseline characteristics are reported for participants with valid data for the respective outcome (Table 1).

**TABLE 1:** Participant characteristics (analytical sample). Female n=18 , male n=19 . Values are mean ± SD for participants with valid baseline data.

Variable	Sex	Mean ± SD	n	p-value (M vs. F)
Age (y)	Female	22.1±2.3	18	0.016
	Male	24.5±3.4	19	
Height (cm)	Female	168.2±4.7	18	< 0.0001
	Male	180.5±7.5	19	
Body mass (kg)	Female	58.7±6.3	18	< 0.0001
	Male	74.3±9.9	19	
Body surface area $\left[{\text{m}}^{2}\right]$	Female	1.67±0.10	18	< 0.0001
	Male	1.94±0.15	19	

p values from Welch’s t-test (two-tailed). The analytical sample includes participants with valid baseline measurements for the respective variables. All participants satisfied the 18–30-year age inclusion criterion and were confirmed to be adults at enrollment. The total number of enrolled participants was 44; exclusions were due to insufficient ultrasound image quality or missing data.

### 2.1 Handling of missing and excluded data

Seven participants were excluded from the analysis of optic nerve sheath diameter due to insufficient ultrasound image quality or missing key data precluding reliable quantification. In addition, seven participants did not complete the full LBNP protocol due to presyncopal symptoms; however, these participants were retained in the analysis, and all available measurements were included. Observations from uncompleted experimental conditions were treated as missing.

### 2.2 Study design and LBNP protocol

All experiments were performed in a quiet, temperature-controlled laboratory (23–25 °C, 50%–55% relative humidity) between 09:00 and 13:00 to minimize circadian influences on cardiovascular regulation. Upon arrival, participants rested in the supine position while the experimental procedures were explained and instrumentation for physiological monitoring was applied.

**FIGURE 2:**
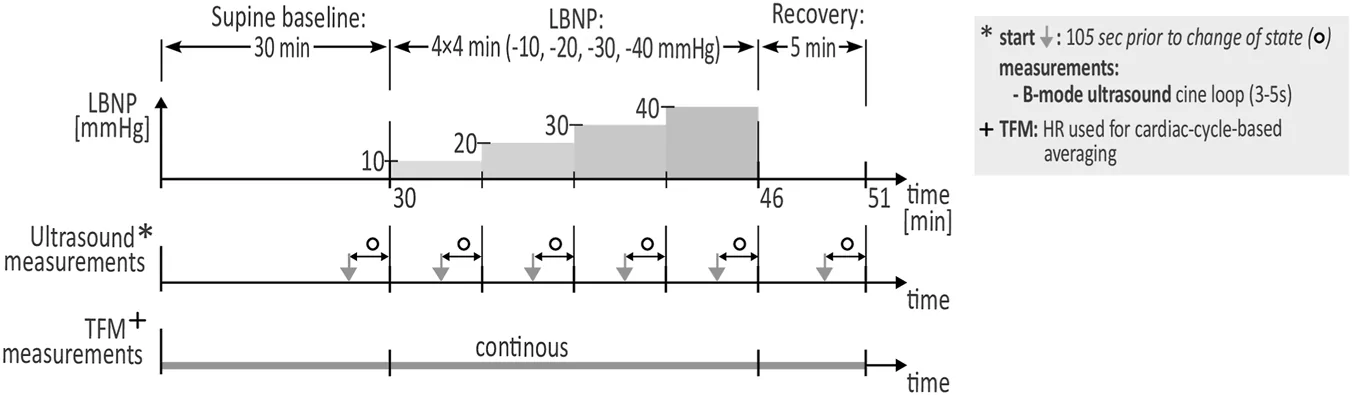
Experimental protocol and timing of measurements. Cardiovascular variables were recorded continuously using a Task Force Monitor (TFM) throughout the experiment. Ultrasound measurements of the optic nerve sheath were performed at discrete time points during supine baseline, graded lower body negative pressure (LBNP; −10 to −40 mmHg in 4-min steps), and recovery. For each condition, short B-mode cine loops (3–5 s) were acquired, and three representative frames were selected for ONSD analysis. Ultrasound acquisition was initiated approximately 105 s prior to each change in experimental condition.

The experimental protocol consisted of three consecutive phases (Figure 2): (i) a 30-min baseline period in the supine position; (ii) graded LBNP exposure lasting 16 min; and (iii) a 5-min recovery period in the supine position. LBNP was initiated at −10 mmHg and increased in steps of 10 mmHg every 4 min up to −40 mmHg. This level of negative pressure was selected because it induces central hypovolemia comparable to that occurring during upright posture and is generally well tolerated in healthy individuals (Cooke et al., 2004; Wolthuis et al., 1974; Hinojosa-Laborde et al., 2014). LBNP was terminated immediately if presyncopal criteria were observed, including marked reductions in arterial blood pressure, light-headedness, visual disturbances, nausea, or at the participant’s request.

### 2.3 Data acquisition

Cardiovascular variables were recorded continuously throughout the protocol using a Task Force® Monitor (TFM; model TFM 3040i, CNSystems, Graz, Austria), providing beat-to-beat measurements of heart rate, arterial blood pressure, stroke volume, cardiac output, and thoracic impedance. In parallel, transbulbar ultrasound measurements of the optic nerve sheath were performed at predefined time points to assess the optic nerve sheath diameter (Figure 2). Ultrasound acquisition was initiated approximately 105 s prior to each change in experimental condition to allow stabilization of hemodynamic responses.

Transbulbar ONSD ultrasound was performed on the right eye in all participants, with the participant supine, the eye closed, and the head in a neutral position. A generous layer of acoustic gel was applied to the closed upper eyelid, and the probe was held with light contact and without any pressure on the globe, in accordance with the technique established in earlier validation studies (Geeraerts et al., 2007; Soldatos et al., 2009). Acoustic output was kept within the U.S. Food and Drug Administration ophthalmic safety limit (mechanical index < 0.23, spatial peak temporal average intensity < 50 mW/ ${cm}^{2}$ ) and following the ALARA principle (Soldatos et al., 2009). All ultrasound acquisition and the subsequent offline ONSD measurements were performed by the same experienced operator with prior methodological work on the transbulbar ONSD ultrasound technique (Bergauer et al., 2012).

#### 2.3.1 Optic nerve sheath diameter measurement

Optic nerve sheath diameter was assessed from B-mode transbulbar ultrasound recordings of the closed right eye using a Philips CX50 ultrasound system (Philips Healthcare, Andover, MA, United States) equipped with a Philips L12-3 broadband linear array transducer. The probe was oriented transversely across the upper eyelid to produce an axial image of the retrobulbar optic nerve. For each experimental condition, short ultrasound cine loops (3–5 s) were acquired, from which three representative frames with clear visualization of the optic nerve sheath were selected for analysis.

**FIGURE 3:**
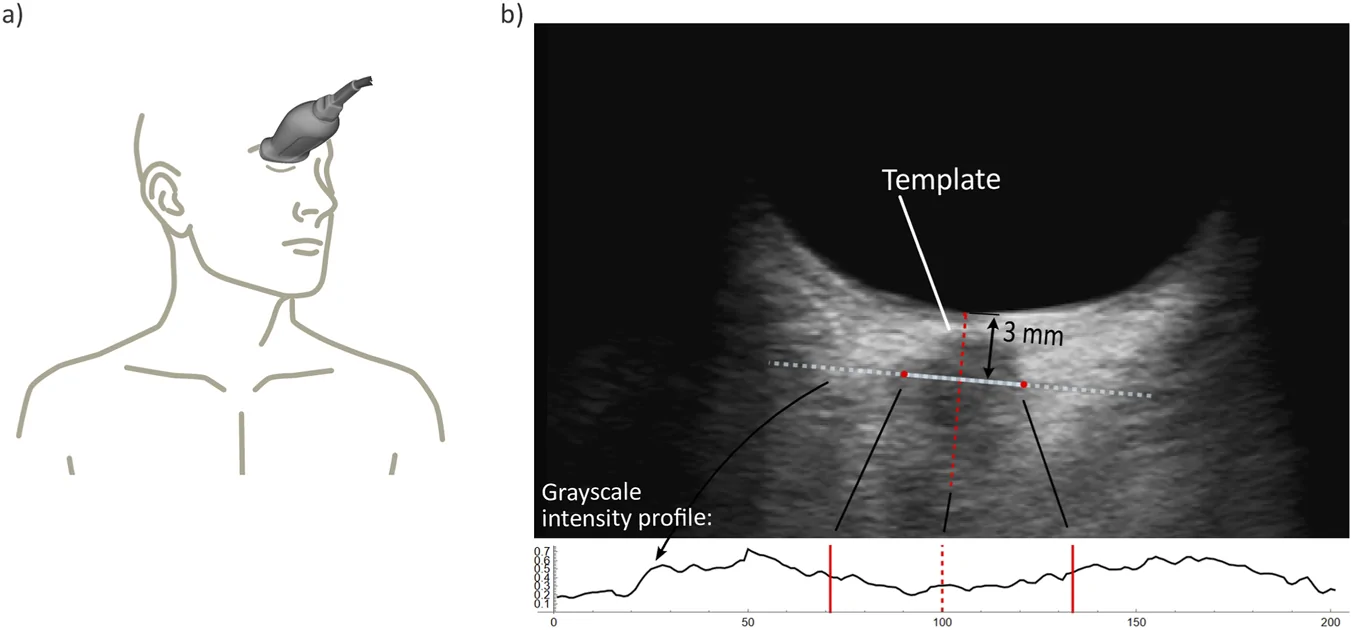
Optic nerve sheath diameter (ONSD) measurement procedure. **(a)** Schematic illustration of ultrasound probe positioning for imaging of the right eye, with the eyelid closed and a layer of acoustic gel between the eyelid and the linear array transducer; no pressure was applied to the globe. **(b)** Representative B-mode ultrasound image with the standardized measurement template aligned with the optic nerve axis. The ONSD is measured along a line positioned 3 mm posterior to the globe, perpendicular to the optic nerve axis. The grayscale intensity profile along the measurement line is shown below to aid identification of optic nerve sheath boundaries.

ONSD measurements were performed using a custom-developed image analysis tool implementing a standardized measurement template (Figure 3). The template was positioned at the posterior boundary of the globe and rotated to align with the optic nerve axis. The optic nerve sheath diameter was measured along a line positioned 3 mm posterior to the globe, perpendicular to the optic nerve axis, between the outer borders of the hypoechoic optic nerve sheath (corresponding to the dural vagina externa shown in Figure 1), in accordance with the protocol established by Helmke and Hansen (1996) and Newman et al. (2002) and widely adopted in subsequent validation studies (Geeraerts et al., 2007; Soldatos et al., 2008; Soldatos et al., 2009; Dubourg et al., 2011; Robba et al., 2018). The grayscale intensity profile along the measurement line was used to aid identification of the optic nerve sheath boundaries (Figure 3b).

The mean ONSD for each experimental condition was computed by averaging measurements across the three selected frames and used in subsequent analyses. Individual ONSD measurements for all 37 analytical-sample participants across the six experimental conditions are provided as Supplementary Table S1.

### 2.4 Statistical analysis

The present work was an exploratory analysis of ONSD data from the parent cohort. The primary outcome was the ONSD dose-response to graded LBNP, whereas the comparison between sexes was a secondary objective of the parent study. No *a priori* sample-size calculation was performed; the sample size was determined by the parent study. To contextualize the secondary sex comparison, we additionally performed a *post hoc* sensitivity power analysis, quantifying the smallest between-sex difference in the LBNP-induced ONSD response that the design could detect, computed from the observed interaction standard errors as $\left(z_{1-\alpha /2}+z_{1-\beta }\right)\times SE$ at 80% statistical power and a two-sided α of 0.05.

Data were analyzed using linear mixed-effects models (LMM) to account for the repeated-measures structure and inter-individual variability. Experimental State (Baseline, LBNP10, LBNP20, LBNP30, LBNP40, Recovery) and Sex (Female, Male) were specified as fixed effects, including their interaction, with participant included as a random intercept. Models were fitted using restricted maximum likelihood estimation. Fixed effects were evaluated using Wald tests; estimated effects are reported as regression coefficients (β) with standard errors (SE). The random-intercept variance was estimated at the parameter-space boundary (singular fit), indicating negligible between-subject variability in the proportional response; fixed-effect estimates and their standard errors were unaffected.

For each participant, ONSD was expressed as a relative change with respect to individual supine baseline ( $\Delta ={ONSD}_{\text{state}}/{ONSD}_{\text{baseline}}-1$ , where Δ=0 at baseline). The distribution of the resulting relative change values was approximately symmetric (skewness ≈0.09 ); logarithmic and Yeo–Johnson transformations did not improve residual normality in exploratory analyses. Data were therefore analyzed on the raw relative-change scale to preserve direct interpretability of coefficients as proportional changes from baseline.

Female participants served as the reference sex category; consequently, State coefficients represent the LBNP-induced change from baseline within the female group, while State × Sex[Male] interaction terms quantify any additional difference in male responses relative to females. Male-specific effects from baseline were additionally assessed by computing linear contrasts (State + State × Sex[Male]) with corresponding standard errors derived from the model covariance matrix.

To assess whether the ONSD response tracked the concurrent systemic hemodynamic response, the relative change in ONSD was related to the relative changes in stroke volume, cardiac output, and heart rate across the four LBNP levels using repeated-measures correlation (Bakdash and Marusich, 2017), which accounts for the within-participant structure of the data; ordinary Pearson correlations are additionally reported in the Supplementary Material.

Model assumptions were assessed by visual inspection of residual and quantile–quantile plots and by the Shapiro–Wilk test. Residuals showed a mild but statistically detectable deviation from normality (Shapiro–Wilk W = 0.977, p = 0.001); this was anticipated given the sample size (211 observations) and was deemed acceptable as linear mixed-effects models with this number of observations are robust to moderate deviations from residual normality. Baseline sex differences in participant characteristics and ONSD were assessed using Welch’s two-sample t-tests for descriptive purposes only and were not included in the mixed-effects models. Statistical significance was set at p < 0.05. All analyses were performed using Python-based statistical packages (statsmodels, pingouin, scipy, seaborn).

### 2.5 Sensitivity analysis (GEE)

As a sensitivity analysis, generalized estimating equations (GEE) with an exchangeable working correlation structure and cluster-robust standard errors were fitted. Results were qualitatively identical to those obtained with the linear mixed-effects model. Full GEE results are provided in Supplementary Table S2.

In an additional sensitivity analysis, the mixed-effects model was refitted after excluding the participants who terminated the protocol early because of presyncopal symptoms (31 participants remaining). The State effects were essentially unchanged, and the significance of every LBNP-induced reduction was preserved (Supplementary Table S4).

No correction for multiple comparisons was applied, as the analyses were structured around a pre-specified primary outcome (ONSD). Interaction terms and secondary contrasts should be interpreted with appropriate caution, particularly given the exploratory nature of sex-specific subgroup analyses.

## 3 Results

Participant characteristics are summarized in Table 1. Men were significantly taller, heavier, and had a greater body surface area than women (all p < 0.0001), and were also slightly older (p = 0.016). No adverse events beyond presyncopal symptoms occurred.

Baseline ONSD values are presented in Table 2. At baseline, ONSD did not differ significantly between sexes (female: 5.21±0.62 mm, n=18 ; male: 5.46±0.70 mm, n=19 ; p = 0.252). The effective analytical sample for the mixed-effects model was 37 participants (18 female, 19 male); seven enrolled participants had no valid ONSD data at any condition and were therefore excluded.

**TABLE 2:** Baseline ONSD values.

Variable	Sex	Mean ± SD	n	p-value (M vs. F)
ONSD [mm]	Female	5.21±0.62	18	0.252
	Male	5.46±0.70	19	

Values represent baseline (supine) measurements. p-value from Welch’s t-test (two-tailed).

### 3.1 ONSD response to graded LBNP

**FIGURE 4:**
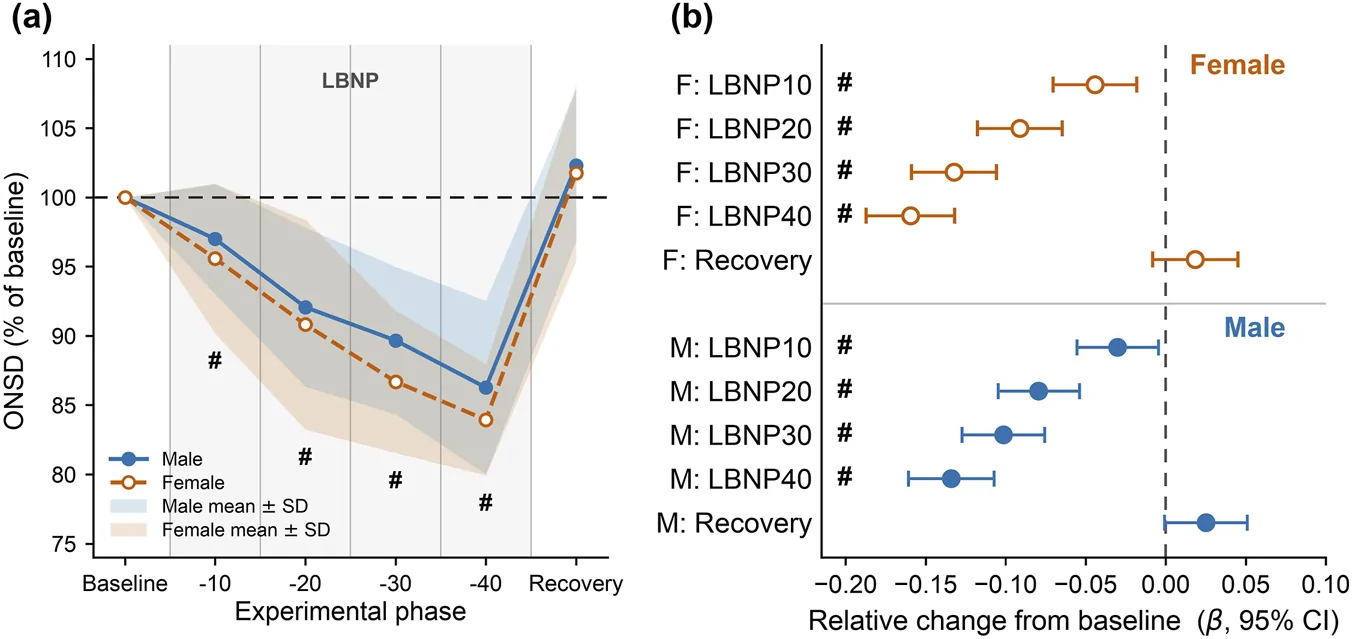
ONSD response to LBNP. **(a)** Group mean ONSD (expressed as % of baseline) during progressive central hypovolemia ( −10 to −40 mmHg LBNP) and recovery. Blue solid lines with filled markers denote males; orange dashed lines with open markers denote females; the surrounding shaded band of the same color shows the mean ± SD for each sex. The shaded background marks the LBNP sphase, with thin vertical lines delimiting the four LBNP levels ( −10 to −40 mmHg). Both sexes showed a progressive, dose-dependent reduction in ONSD with increasing LBNP intensity. The hash symbol (#) indicates a statistically significant change from baseline based on the linear mixed-effects model (p < 0.05). **(b)** Forest plot of LMM coefficients on the raw relative-change scale, using the same colors as panel **(a)**. Orange open circles show female-reference State effects ( β± 95% CI); blue filled circles show male-specific contrasts computed as State + State × Sex[Male]. The vertical dashed line marks no effect (β=0) . Intervals entirely to the left of zero indicate a statistically significant ONSD reduction relative to baseline (p < 0.05, marked by #). Because the analysis is on the raw relative-change scale, β values map directly to proportional changes (e.g., β=−0.159 corresponds to −15.9% at LBNP40). The full fixed-effect table, including State × Sex[Male] interaction coefficients, is provided in Table 3. Data are derived from the right eye.

A strong main effect of State was observed, with ONSD progressively decreasing as LBNP intensity increased (Figure 4). Coefficients β represent the relative change in ONSD compared with baseline (i.e., ${ONSD}_{\text{state}}/{ONSD}_{\text{baseline}}-1$ ).

#### 3.1.1 Female-specific LBNP effects

Female participants showed statistically significant reductions in ONSD at every LBNP level compared with baseline: LBNP10 ( β=−0.044±0.013 , p = 0.001; approximately −4.4% ), LBNP20 ( β=−0.091±0.014 , p < 0.001; approximately −9.1% ), LBNP30 ( β=−0.132±0.014 , p < 0.001; approximately −13.2% ), and LBNP40 ( β=−0.159±0.014 , p < 0.001; approximately −15.9% ). During recovery, ONSD returned toward baseline ( β=+0.018±0.014 , p = 0.173).

#### 3.1.2 Male-specific LBNP effects

Male participants showed similar reductions at all LBNP levels, derived as linear contrasts from the full mixed model: LBNP10 ( Δ=−0.030±0.013 , p = 0.022), LBNP20 ( Δ=−0.079±0.013 , p < 0.001), LBNP30 ( Δ=−0.101±0.013 , p < 0.001), and LBNP40 ( Δ=−0.134±0.014 , p < 0.001). A borderline non-significant increase in ONSD was observed during recovery ( Δ=+0.025±0.013 , p = 0.059). Group means of absolute ONSD (in mm) at each state, by sex, are provided in Supplementary Table S3 and show the same monotonic reduction in both sexes, from 5.21±0.62 mm (female) and 5.46±0.70 mm (male) at baseline to 4.31±0.49 mm and 4.76±0.48 mm, respectively, at LBNP40.

#### 3.1.3 Sex effects and state × sex interactions

The main effect of sex was not significant (p = 1.00); no statistically significant difference in overall ONSD response was detected between male and female participants. No state × sex interaction reached statistical significance (all p > 0.1; see Table 3), suggesting that the relative reduction in ONSD during LBNP was broadly similar between sexes. The state × sex interaction estimates were small, ranging from +0.7 to +3.1 percentage points (Table 3). In a *post hoc* sensitivity analysis, given the observed interaction standard errors, the study had 80% power to detect a between-sex difference in the LBNP-induced ONSD change of approximately 5 percentage points (two-sided α = 0.05); all observed differences were below this threshold, so that only smaller sex-specific effects could have gone undetected.

**TABLE 3:** Fixed-effect estimates from the linear mixed-effects model for ONSD. The response variable is the relative change from individual baseline $\left({ONSD}_{\text{state}}/{ONSD}_{\text{baseline}}-1\right)$ . State coefficients represent the effect of LBNP within the female reference group; State × Sex[Male] interactions represent additional differences for males. Wald test p-values; significant effects in bold (p < 0.05). The random-intercept variance was estimated near zero (singular fit); the likelihood-ratio p-value for this variance component is therefore not estimable, and the model is effectively equivalent to a fixed-effects repeated-measures regression.

Fixed effect	Estimate (β)	p-value
Intercept	+0.000	1.000
State [LBNP10]	−0.044	0.001
State [LBNP20]	−0.091	<0.001
State [LBNP30]	−0.132	<0.001
State [LBNP40]	−0.159	<0.001
State [recovery]	+0.018	0.173
Sex [male]	−0.000	1.000
State [LBNP10] × sex [male]	+0.014	0.447
State [LBNP20] × sex [male]	+0.012	0.532
State [LBNP30] × sex [male]	+0.031	0.103
State [LBNP40] × sex [male]	+0.025	0.195
State [recovery] × sex [male]	+0.007	0.728
Group Var (random intercept)	0.001	≈0 (singular fit)

Model: $\left({ONSD}_{\text{state}}/{ONSD}_{\text{baseline}}\right)-1∼\text{State}\times \text{Sex}+\left(1\;|\text{ Subject}\right)$ . Note on coefficients: State[x] = female-specific LBNP effect; State[x] × Sex[Male] = additional male deviation. Male-specific contrasts (State + State × Sex[Male]) are reported separately in the text. Coefficients can be interpreted directly as proportional changes from baseline.

#### 3.1.4 Association with the systemic hemodynamic response

The relative reduction in ONSD paralleled the concurrent hemodynamic response to LBNP. Across the four LBNP levels, the relative change in ONSD was positively correlated with the relative changes in stroke volume $\left(r_{\text{rm}}=0.64\right)$ and cardiac output $\left(r_{\text{rm}}=0.48\right)$ and negatively correlated with the relative change in heart rate ( $r_{\text{rm}}=-0.53$ ; all p < 0.001, repeated-measures correlation; Supplementary Table S5).

Results from the full mixed-effects model are presented in Table 3. Mean ± 95% CI of ONSD changes during progressive LBNP and recovery are illustrated in Figure 4.

## 4 Discussion

The present study shows that progressive LBNP induces a graded, dose-dependent reduction in ONSD that is statistically significant from −10 mmHg onward and reaches approximately − 15% of baseline at −40 mmHg.

Importantly, the magnitude of this response is not only statistically significant but also physiologically substantial. Given a baseline ONSD of approximately 5.3 mm, a 15% reduction corresponds to an absolute decrease of roughly 0.8 mm, which is of similar order to ONSD differences reported between clinically distinct intracranial-pressure states in previous ultrasound studies. Although ONSD cannot be translated directly into absolute ICP values, the observed effect size suggests that graded LBNP produces a robust physiological alteration in the intracranial compartment rather than a merely marginal measurement change.

There were no statistically significant differences in responses between female and male participants, and no state × sex interaction reached significance. This graded ONSD dose-response to mild and moderate LBNP in the supine position in a mixed-sex cohort of healthy adults complements earlier observations made under head-down tilt conditions (Marshall-Goebel et al., 2017b; Watkins et al., 2017).

The observed reduction in ONSD with progressive LBNP is consistent with a CVP-mediated reduction in ICP. During LBNP, blood pooling in the lower extremities reduces thoracic and central venous pressure (Cooke et al., 2004; Wolthuis et al., 1974; Hinojosa-Laborde et al., 2014; Goswami et al., 2019a; Goswami, 2023). According to the Davson model of CSF dynamics, ICP is governed by the balance between CSF formation and outflow against the dural venous-sinus pressure, such that reductions in CVP are expected to lower dural venous-sinus pressure and thereby reduce ICP. Empirical support for this proposed coupling was recently provided by Hansen et al. (2021), who showed that a non-pharmacological intervention lowering CVP by ∼ 3 mmHg simultaneously reduced invasively measured ICP by ∼ 4 mmHg, with Δ CVP and Δ ICP linearly correlated across subjects ( ${\text{R}}^{2}$ = 0.77). The progressive ONSD reductions observed here are consistent with this proposed coupling between CVP and ICP: as perioptic CSF pressure falls, the retrobulbar sheath undergoes a passive reduction in diameter. This interpretation is supported by magnetic-resonance observations showing that ICP correlates with ONSD rather than with the diameter of the optic nerve itself (Geeraerts et al., 2008).

These findings agree with two complementary lines of prior evidence. First, the only previous study of LBNP and ONSD in healthy volunteers showed that −20 mmHg LBNP attenuated the ONSD distention produced by −12° head-down tilt (Marshall-Goebel et al., 2017b); the present data extend that observation to the supine posture, in the absence of any cephalad shift to oppose it, and Watkins et al. (2017) similarly reported LBNP-induced reductions in non-invasively measured ICP and internal jugular vein cross-sectional area. Second, manipulations acting in the opposite direction produce the opposite response: Trendelenburg positioning, which raises central venous pressure and impairs cerebral venous drainage, increases ONSD, an effect reversed by head-of-bed elevation (Maissan et al., 2018). Together, these observations support ONSD as a sensitive marker of pressure-related changes within the craniospinal system, detectable even for the relatively small shifts seen in longitudinal high-altitude studies (Sutherland et al., 2008; Fagenholz et al., 2009).

Within this framework, whether men and women respond differently is of particular physiological interest. The absence of a significant main sex effect or of significant state × sex interactions indicates that the present study did not detect differential ONSD responses to graded central hypovolemia between the sexes. This is noteworthy given well-documented sex differences in autonomic regulation and orthostatic tolerance, with females typically exhibiting a greater heart-rate response and lower tolerance to high-grade LBNP (Convertino, 1998; Evans et al., 2018; Goswami et al., 2019a; Goswami et al., 2021; Goswami et al., 2026b; Goswami et al., 2026a). Indeed, in the same cohort, systemic responses to the identical protocol did differ by sex: in heart-rate and stroke-index dynamics (Shankhwar et al., 2023), in modeled vascular-compensation strategies (Bergauer et al., 2026), and in neurohormonal fluid-regulating effectors (Goswami et al., 2019b). That these systemic differences did not translate into detectable ONSD differences suggests that the mechanical CVP–ICP coupling predominates over sex-specific compensation during supine central hypovolemia. Because this *post hoc* sensitivity analysis indicated that the design could detect only a between-sex difference of approximately 5 percentage points in the LBNP-induced ONSD response, smaller sex-specific effects cannot be excluded. From a network-physiology standpoint, the sex-divergent outputs of several interacting subsystems (autonomic, cardiac, vascular, and neurohormonal) converge onto a common cerebro-ocular pressure node, so that the mechanical CVP–ICP coupling dominates the integrated ONSD readout despite this upstream heterogeneity. Consistent with this integrated view, the magnitude of the ONSD reduction tracked the concurrent fall in stroke volume and cardiac output and the rise in heart rate within the same participants (Supplementary Table S5), directly linking the cerebro-ocular readout to the systemic hemodynamic response. Notably, the only prior report of sex-related ONSD differences in healthy adults under physiological stress attributed the ∼ 1 mm smaller female ONSD to body height rather than to sex *per se* (Sutherland et al., 2008).

ONSD returned toward baseline during recovery in both sexes; the borderline overshoot in males (p = 0.059) may reflect a transient post-LBNP rebound in central venous return, but its small magnitude (+2.5% of baseline) and absence in females preclude firm interpretation. The rapidity of this normalization, within a 5-min recovery window, is consistent with previous observations that ONSD tracks acute changes in ICP within seconds to minutes (Moretti et al., 2009), providing additional support for the use of ONSD as a dynamic, repeated-measures marker of acute ICP-related changes rather than only as a static threshold diagnostic.

The clinical and translational relevance of these findings lies primarily in the context of SANS, in which microgravity-induced cephalad fluid redistribution causes sustained elevations of ICP, ONSD distention, optic-disc edema, globe flattening, and choroidal folds (Mader et al., 2011; 2021; Lee et al., 2020; Martin Paez et al., 2020; Stenger et al., 2019). LBNP has been proposed as a mechanical countermeasure because it acutely reduces ICP, attenuates head-down-tilt-induced increases in intraocular pressure and ICP, and reduces ONSD (Marshall-Goebel et al., 2017b; Marshall-Goebel et al., 2017a; Watkins et al., 2017; Petersen et al., 2019; Stenger et al., 2019). Additional indirect evidence supporting a potential protective role of LBNP comes from observations that Russian cosmonauts routinely using LBNP-based countermeasures exhibit less microgravity-induced perivascular-space enlargement than astronauts not using such protocols (Wostyn et al., 2022). The translation of LBNP into operational countermeasure use, however, must also be weighed against the risk of venous stasis and lower-limb thrombosis associated with prolonged or high-grade LBNP exposure (Harris et al., 2022; Marshall-Goebel et al., 2019). Our finding that even mild LBNP ( −10 mmHg) produced a measurable reduction in ONSD suggests that the relevant CVP-to-ICP coupling may already be engaged at relatively low levels of negative pressure, a finding potentially relevant to the development of low-intensity SANS countermeasures.

Beyond spaceflight physiology, the ability of transbulbar ultrasound to detect reductions in ONSD may have translational relevance for clinical intracranial hypotension. Intracranial hypotension, whether spontaneous or iatrogenic (for example, following spinal anesthesia or an inadvertent dural breach during epidural analgesia), remains a clinically important and underexplored condition whose diagnosis frequently relies on magnetic resonance imaging (Schievink, 2006). As an illustrative perspective, a non-invasive reduction in ONSD could conceivably serve as a bedside reference in this setting, for example, as a baseline value that normalizes after an epidural blood patch and could be re-assessed upon symptom recurrence. Consistent with this idea, preliminary clinical work has shown that transorbital ONSD ultrasonography can discriminate spontaneous intracranial hypotension and that the optic nerve sheath diameter changes after microsurgical or epidural treatment of the underlying cerebrospinal-fluid leak (Fichtner et al., 2016; Fichtner et al., 2019). Given the limited literature on non-invasive approaches to intracranial hypotension, this avenue may warrant dedicated investigation. This possibility is offered as a hypothesis to be tested rather than as a conclusion supported by the present data.

These findings indicate that supine LBNP produces a robust, graded reduction in ONSD, consistent with the proposed coupling between CVP and ICP and supporting the use of ONSD ultrasound as a sensitive marker of acute ICP-related changes. Within the present cohort, the magnitude and pattern of the response were similar in males and females, although the study was not specifically powered to establish equivalence; therefore, smaller sex-specific effects cannot be excluded.

### 4.1 Limitations

Several limitations should be acknowledged. First, ONSD is an indirect surrogate marker of ICP, and neither ICP nor CVP were measured directly. Consequently, the proposed mechanism whereby reductions in CVP lower ICP and thereby reduce ONSD relies on evidence from previous invasive studies (Hansen et al., 2021; Petersen et al., 2019; Geeraerts et al., 2007; Soldatos et al., 2008; Geeraerts et al., 2008) rather than direct verification in the present cohort. Moreover, ONSD does not reflect ICP alone: local determinants such as the compliance and trabecular architecture of the optic nerve sheath and the dynamics of the perioptic subarachnoid space also modulate this relationship, so that changes in ONSD are best interpreted as consistent with, rather than direct measures of, changes in ICP. Relatedly, symptomatic correlates of intracranial hypotension, such as headache, were not systematically recorded during the protocol, in which only presyncopal criteria were monitored for participant safety; documenting such symptoms alongside the ONSD reduction would have strengthened the mechanistic interpretation and should be considered in future studies. Second, ONSD measurements were limited to the right eye. Although ONSD responses to ICP changes are generally bilateral (Newman et al., 2002; Soldatos et al., 2008), unilateral measurements may not fully capture inter-ocular variability. Third, ONSD was derived from three representative frames per condition and therefore does not capture within-condition temporal variability; ultrasound-based ONSD measurements are also subject to measurement uncertainty and operator variability (Karakitsos et al., 2006; Geeraerts et al., 2007; Soldatos et al., 2008; Moretti et al., 2009; Geeraerts et al., 2008). In addition, all ultrasound measurements were performed by a single experienced operator; although this maximizes internal consistency, inter-observer variability was not assessed in the present study (Ballantyne et al., 2002). Fourth, the menstrual-cycle phase of female participants was not controlled, although oral contraceptive use, a further potential source of hormonal variability, can be excluded, since none of the female participants was using hormonal contraception (see Materials and Methods). Previous studies have demonstrated menstrual-phase effects on cardiovascular and orthostatic responses to LBNP (Goswami et al., 2020; Shankhwar et al., 2024). Although no significant sex-related differences in ONSD response were detected in the present study, a potential contribution of hormonal status to smaller ONSD effects cannot be excluded and warrants investigation in future studies. Finally, the experiment simulated central hypovolemia in the supine position; results may not directly extrapolate to upright posture or to the sustained cephalad fluid shifts of microgravity, where the opposite gradient applies. The implications for SANS countermeasure design therefore remain hypothesis-generating.

## 5 Conclusion

This study shows that graded LBNP induces a progressive, dose-dependent reduction in ONSD in healthy young adults, detectable at −10 mmHg and increasing with LBNP intensity up to −40 mmHg. No significant sex-related differences were observed, consistent with a mechanical response to central hypovolemia. ONSD returned toward baseline within 5 minutes of LBNP termination, supporting its utility as a dynamic, non-invasive marker of acute ICP-related changes. These findings extend current evidence supporting ONSD ultrasound as a sensitive tool for monitoring physiological responses to altered central fluid distribution and may have relevance for the development and evaluation of countermeasures targeting microgravity-induced cephalad fluid shifts.

## Data Availability

The original contributions, including the individual ONSD data and all analyses, are included in the article and Supplementary Material.

## References

[B1] Aspide R. Bertolini G. Albini Riccioli L. Mazzatenta D. Palandri G. Biasucci D. (2020). A proposal for a new protocol for sonographic assessment of the optic nerve sheath diameter: the closed protocol. Neurocrit. Care 32, 327–332. 10.1007/s12028-019-00853-x 31583527

[B2] Bakdash J. Marusich L. (2017). Repeated measures correlation. Front. Psychol. 8, 456. 10.3389/fpsyg.2017.00456 28439244 PMC5383908

[B3] Ballantyne S. O’Neill G. Hamilton R. Hollman A. (2002). Observer variation in the sonographic measurement of optic nerve sheath diameter in normal adults. Eur. J. Ultrasound 15, 145–149. 10.1016/S0929-8266(02)00036-8 12423741

[B4] Bashan A. Bartsch R. Kantelhardt J. Havlin S. Ivanov P. (2012). Network physiology reveals relations between network topology and physiological function. Nat. Commun. 3, 702. 10.1038/ncomms1705 22426223 PMC3518900

[B5] Bergauer A. Prosen G. Flis V. Šeruga T. Brvar M. Kobilica N. (2012). Contrast enhanced ultrasound imaging of the optic nerve sheath diameter – what are we really measuring? Crit. Ultrasound J. 4, A2. 10.1186/2036-7902-4-S1-A2

[B6] Bergauer A. Prosen G. Kobilica N. Flis V. Jevšek M. Šeruga T. (2017). Contrast-enhanced ultrasound imaging of the optic nerve sheath diameter – a proof of concept study. Acta Medico-Biotechnica 10, 25–33. 10.18690/actabiomed.151

[B7] Bergauer A. Urevc J. Halilović M. Batzel J. Pivec V. Goswami N. (2026). Modeling sex-dependent cardiovascular responses to lower body negative pressure. Am. J. Physiol. Heart Circ. Physiol. 331, H240–H259. 10.1152/ajpheart.00258.2026 42213715

[B8] Blaivas M. Theodoro D. Sierzenski P. (2003). Elevated intracranial pressure detected by bedside emergency ultrasonography of the optic nerve sheath. Acad. Emerg. Med. 10, 376–381. 10.1111/j.1553-2712.2003.tb01352.x 12670853

[B9] Convertino V. (1998). Gender differences in autonomic functions associated with blood pressure regulation. Am. J. Physiol. Regul. Integr. Comp. Physiol. 275, R1909–R1920. 10.1152/ajpregu.1998.275.6.R1909 9843880

[B10] Cooke W. Ryan K. Convertino V. (2004). Lower body negative pressure as a model to study progression to acute hemorrhagic shock in humans. J. Appl. Physiol. 96, 1249–1261. 10.1152/japplphysiol.01155.2003 15016789

[B11] Dubourg J. Javouhey E. Geeraerts T. Messerer M. Kassai B. (2011). Ultrasonography of optic nerve sheath diameter for detection of raised intracranial pressure: a systematic review and meta-analysis. Intensive Care Med. 37, 1059–1068. 10.1007/s00134-011-2224-2 21505900

[B12] Evans J. Knapp C. Goswami N. (2018). Artificial gravity as a countermeasure to the cardiovascular deconditioning of spaceflight: gender perspectives. Front. Physiol. 9, 716. 10.3389/fphys.2018.00716 30034341 PMC6043777

[B13] Fagenholz P. Gutman J. Murray A. Noble V. Camargo C. Harris N. (2009). Optic nerve sheath diameter correlates with the presence and severity of acute mountain sickness: evidence for increased intracranial pressure. J. Appl. Physiol. 106, 1207–1211. 10.1152/japplphysiol.01188.2007 19118159

[B14] Fichtner J. Ulrich C. Fung C. Knüppel C. Veitweber M. Jilch A. (2016). Management of spontaneous intracranial hypotension – transorbital ultrasound as discriminator. J. Neurol. Neurosurg. Psychiatry 87, 650–655. 10.1136/jnnp-2015-310853 26285586 PMC4893146

[B15] Fichtner J. Ulrich C. Fung C. Cipriani D. Gralla J. Piechowiak E. (2019). Sonography of the optic nerve sheath diameter before and after microsurgical closure of a dural csf fistula in patients with spontaneous intracranial hypotension – a consecutive cohort study. Cephalalgia 39, 306–315. 10.1177/0333102418793640 30099952

[B16] Geeraerts T. Launey Y. Martin L. Pottecher J. Vigué B. Duranteau J. (2007). Ultrasonography of the optic nerve sheath may be useful for detecting raised intracranial pressure after severe brain injury. Intensive Care Med. 33, 1704–1711. 10.1007/s00134-007-0797-6 17668184

[B17] Geeraerts T. Newcombe V. Coles J. Abate M. Perkes I. Hutchinson P. (2008). Use of T2-weighted magnetic resonance imaging of the optic nerve sheath to detect raised intracranial pressure. Crit. Care 12, R114. 10.1186/cc7006 18786243 PMC2592740

[B18] Goswami N. (2023). Compensatory hemodynamic changes in response to central hypovolemia in humans: lower body negative pressure: updates and perspectives. J. Muscle Res. Cell Motil. 44, 89–94. 10.1007/s10974-022-09635-z 36380185 PMC10329599

[B19] Goswami N. Blaber A. Hinghofer-Szalkay H. Convertino V. (2019a). Lower body negative pressure: physiological effects, applications, and implementation. Physiol. Rev. 99, 807–851. 10.1152/physrev.00006.2018 30540225

[B20] Goswami N. Reichmuth J. Di Mise A. Brix B. Roessler A. Centrone M. (2019b). Comparison between men and women of volume regulating hormones and aquaporin-2 excretion following graded central hypovolemia. Eur. J. Appl. Physiol. 119, 633–643. 10.1007/s00421-018-4053-2 30564880

[B21] Goswami N. Brix B. Roessler A. Koestenberger M. Reibnegger G. Cvirn G. (2020). Menstrual phase affects coagulation and hematological parameters during central hypovolemia. J. Clin. Med. 9, 3118. 10.3390/jcm9103118 32992471 PMC7600806

[B22] Goswami N. White O. Blaber A. Evans J. van Loon J. Clement G. (2021). Human physiology adaptation to altered gravity environments. Acta Astronaut. 189, 216–221. 10.1016/j.actaastro.2021.08.023

[B23] Goswami N. Batzel J. Ma Y. Fredriksen P. Green D. Ivanov P. (2026a). Network physiology in space: vision and perspectives on exploring physiological networks during spaceflight for the benefit of life on earth. Front. Netw. Physiol. 6, 1817815. 10.3389/fnetp.2026.1817815 42109464 PMC13152824

[B24] Goswami N. Blaber A. Valenti G. Hinghofer-Szalkay H. Evans J. Bailey D. (2026b). Gravity, microgravity, and artificial gravity: physiological effects, implementation, and applications. Physiol. Rev. 106, 750–839. 10.1152/physrev.00055.2024 41021767

[B25] Hansen A. Lawley J. Rickards C. Howden E. Sarma S. Cornwell W. (2021). Reducing intracranial pressure by reducing central venous pressure: assessment of potential countermeasures to spaceflight-associated neuro-ocular syndrome. J. Appl. Physiol. 130, 283–289. 10.1152/japplphysiol.00786.2020 33270516

[B26] Harris K. Weber T. Greaves D. Green D. Goswami N. Petersen L. (2022). Going against the flow: are venous thromboembolism and impaired cerebral drainage critical risks for spaceflight?. J. Appl. Physiol. 132, 270–273. 10.1152/japplphysiol.00425.2021 34672768 PMC8759966

[B27] Helmke K. Hansen H. (1996). Fundamentals of transorbital sonographic evaluation of optic nerve sheath expansion under intracranial hypertension: experimental study. Pediatr. Radiol. 26, 701–705. 10.1007/BF01383383 8805599

[B28] Hinojosa-Laborde C. Shade R. Muniz G. Bauer C. Goei K. Pidcoke H. (2014). Validation of lower body negative pressure as an experimental model of hemorrhage. J. Appl. Physiol. 116, 406–415. 10.1152/japplphysiol.00640.2013 24356525 PMC4073981

[B29] Ivanov P. (2021). The new field of network physiology: building the human physiolome. Front. Netw. Physiol. 1, 711778. 10.3389/fnetp.2021.711778 36925582 PMC10013018

[B30] Karakitsos D. Soldatos T. Gouliamos A. Armaganidis A. Poularas J. Kalogeromitros A. (2006). Transorbital sonographic monitoring of optic nerve diameter in patients with severe brain injury. Transpl. Proc. 38, 3700–3706. 10.1016/j.transproceed.2006.10.185 17175372

[B31] Lee A. Mader T. Gibson C. Tarver W. Rabiei P. Riascos R. (2020). Spaceflight associated neuro-ocular syndrome (SANS) and the neuro-ophthalmologic effects of microgravity: a review and an update. NPJ Microgravity 6, 7. 10.1038/s41526-020-0097-9 32047839 PMC7005826

[B32] Liu D. Li Z. Zhang X. Zhao L. Jia J. Sun F. (2017). Assessment of intracranial pressure with ultrasonographic retrobulbar optic nerve sheath diameter measurement. BMC Neurol. 17, 188. 10.1186/s12883-017-0964-5 28962603 PMC5622417

[B33] Ly V. Velichala S. Hargens A. (2022). Cardiovascular, lymphatic, and ocular health in space. Life (Basel) 12, 268. 10.3390/life12020268 35207555 PMC8875500

[B34] Mader T. Gibson C. Pass A. Kramer L. Lee A. Fogarty J. (2011). Optic disc edema, globe flattening, choroidal folds, and hyperopic shifts observed in astronauts after long-duration space flight. Ophthalmology 118, 2058–2069. 10.1016/j.ophtha.2011.06.021 21849212

[B35] Mader T. Gibson C. Barratt M. Miller N. Subramanian P. Killer H. E. (2021). Persistent globe flattening in astronauts following long-duration spaceflight. Neuro-Ophthalmology 45, 29–35. 10.1080/01658107.2020.1791189 33762785 PMC7946045

[B36] Maissan I. Verbaan L. van den Berg M. Houmes R. Stolker R. den Hartog D. (2018). Helicopter transportation increases intracranial pressure: a proof-of-principle study. Air Med. J. 37, 249–252. 10.1016/j.amj.2018.02.010 29935704

[B37] Marshall-Goebel K. Mulder E. Bershad E. Laing C. Eklund A. Malm J. (2017a). Intracranial and intraocular pressure during various degrees of head-down tilt. Aerosp. Med. Hum. Perform. 88, 10–16. 10.3357/AMHP.4653.2017 28061916

[B38] Marshall-Goebel K. Terlević R. Gerlach D. Kuehn S. Mulder E. Rittweger J. (2017b). Lower body negative pressure reduces optic nerve sheath diameter during head-down tilt. J. Appl. Physiol. 123, 1139–1144. 10.1152/japplphysiol.00256.2017 28818998

[B39] Marshall-Goebel K. Laurie S. Alferova I. Arbeille P. Auñón-Chancellor S. Ebert D. (2019). Assessment of jugular venous blood flow stasis and thrombosis during spaceflight. JAMA Netw. Open 2, e1915011. 10.1001/jamanetworkopen.2019.15011 31722025 PMC6902784

[B40] Martin Paez Y. Mudie L. Subramanian P. (2020). Spaceflight associated neuro-ocular syndrome (SANS): a systematic review and future directions. Eye Brain 12, 105–117. 10.2147/EB.S234076 33117025 PMC7585261

[B41] Montorfano L. Yu Q. Bordes S. Sivanushanthan S. Rosenthal R. Montorfano M. (2021). Mean value of B-mode optic nerve sheath diameter as an indicator of increased intracranial pressure: a systematic review and meta-analysis. Ultrasound J. 13, 35. 10.1186/s13089-021-00235-5 34215966 PMC8253877

[B42] Moretti R. Pizzi B. Cassini F. Vivaldi N. (2009). Reliability of optic nerve ultrasound for the evaluation of patients with spontaneous intracranial hemorrhage. Neurocrit. Care 11, 406–410. 10.1007/s12028-009-9250-8 19636971

[B43] Newman W. Hollman A. Dutton G. Carachi R. (2002). Measurement of optic nerve sheath diameter by ultrasound: a means of detecting acute raised intracranial pressure in hydrocephalus. Br. J. Ophthalmol. 86, 1109–1113. 10.1136/bjo.86.10.1109 12234888 PMC1771326

[B44] Ogoh S. Hirasawa A. Shibata S. (2022). Influence of head-up tilt and lower body negative pressure on the internal jugular vein. Physiol. Rep. 10, e15248. 10.14814/phy2.15248 35581747 PMC9114655

[B45] Petersen L. Lawley J. Lilja-Cyron A. Petersen J. Howden E. Sarma S. (2019). Lower body negative pressure to safely reduce intracranial pressure. J. Physiol. 597, 237–248. 10.1113/JP276557 30286250 PMC6312426

[B46] Rajajee V. Vanaman M. Fletcher J. Jacobs T. (2011). Optic nerve ultrasound for the detection of raised intracranial pressure. Neurocrit. Care 15, 506–515. 10.1007/s12028-011-9606-8 21769456

[B47] Robba C. Santori G. Czosnyka M. Corradi F. Bragazzi N. Padayachy L. (2018). Optic nerve sheath diameter measured sonographically as non-invasive estimator of intracranial pressure: a systematic review and meta-analysis. Intensive Care Med. 44, 1284–1294. 10.1007/s00134-018-5305-7 30019201

[B48] Schievink W. (2006). Spontaneous spinal cerebrospinal fluid leaks and intracranial hypotension. JAMA 295, 2286–2296. 10.1001/jama.295.19.2286 16705110

[B49] Shankhwar V. Urvec J. Steuber B. Schmid Zalaudek K. Bergauer A. Alsuwaidi H. (2023). Association of gender with cardiovascular and autonomic responses to central hypovolemia. Front. Cardiovasc. Med. 10, 1211774. 10.3389/fcvm.2023.1211774 37719984 PMC10501725

[B50] Shankhwar V. Urvec J. Steuber B. Schmid Zalaudek K. Salon A. Hawliczek A. (2024). Effects of menstrual cycle on hemodynamic and autonomic responses to central hypovolemia. Front. Cardiovasc. Med. 11, 1290703. 10.3389/fcvm.2024.1290703 38361585 PMC10867210

[B51] Soldatos T. Karakitsos D. Chatzimichail K. Papathanasiou M. Gouliamos A. Karabinis A. (2008). Optic nerve sonography in the diagnostic evaluation of adult brain injury. Crit. Care 12, R67. 10.1186/cc6897 18477382 PMC2481450

[B52] Soldatos T. Chatzimichail K. Papathanasiou M. Gouliamos A. (2009). Optic nerve sonography: a new window for the non-invasive evaluation of intracranial pressure in brain injury. Emerg. Med. J. 26, 630–634. 10.1136/emj.2008.058453 19700575

[B53] Steinborn M. Fiegler J. Kraus V. Denne C. Hapfelmeier A. Wurzinger L. (2011). High resolution ultrasound and magnetic resonance imaging of the optic nerve and the optic nerve sheath: anatomic correlation and clinical importance. Ultraschall Med. 32, 608–613. 10.1055/s-0029-1245822 21058238

[B54] Stenger M. Laurie S. Sadda S. Sadun A. Macias B. Huang A. (2019). Focus on the optic nerve head in spaceflight-associated neuro-ocular syndrome. Ophthalmology 126, 1604–1606. 10.1016/j.ophtha.2019.09.009 31759496

[B55] Sutherland A. Morris D. Owen C. Bron A. Roach R. (2008). Optic nerve sheath diameter, intracranial pressure and acute mountain sickness on Mount Everest: a longitudinal cohort study. Br. J. Sports Med. 42, 183–188. 10.1136/bjsm.2007.045286 18182624

[B56] Taneja I. Moran C. Medow M. Glover J. Montgomery L. Stewart J. (2007). Differential effects of lower body negative pressure and upright tilt on splanchnic blood volume. Am. J. Physiol. Heart Circ. Physiol. 292, H1420–H1426. 10.1152/ajpheart.01096.2006 17085534 PMC4517828

[B57] Tayal V. Neulander M. Norton H. Foster T. Saunders T. Blaivas M. (2007). Emergency department sonographic measurement of optic nerve sheath diameter to detect findings of increased intracranial pressure in adult head injury patients. Ann. Emerg. Med. 49, 508–514. 10.1016/j.annemergmed.2006.06.040 16997419

[B58] Watkins W. Hargens A. Seidl S. Clary E. Macias B. (2017). Lower-body negative pressure decreases noninvasively measured intracranial pressure and internal jugular vein cross-sectional area during head-down tilt. J. Appl. Physiol. 123, 260–266. 10.1152/japplphysiol.00091.2017 28495841 PMC5538811

[B59] Wolthuis R. Bergman S. Nicogossian A. (1974). Physiological effects of locally applied reduced pressure in man. Physiol. Rev. 54, 566–595. 10.1152/physrev.1974.54.3.566 4601623

[B60] Wostyn P. Mader T. Gibson C. Nedergaard M. (2022). Does long-duration exposure to microgravity lead to dysregulation of the brain and ocular glymphatic systems? Eye Brain 14, 49–58. 10.2147/EB.S354710 35546965 PMC9081191

