# Supplementary material for: Dose-dependent reduction of optic nerve sheath diameter during graded lower body negative pressure in healthy adults of both sexes
Source: Front Netw Physiol. 2026 Aug 7;6:1908460. doi: 10.3389/fnetp.2026.1908460 (PMC13493247; doi:10.3389/fnetp.2026.1908460)
Supplement: Supplementary file 1 [file DataSheet1.pdf]

Supplementary Material

1 SUPPLEMENTARY TABLES

This supplementary file contains the individual optic nerve sheath diameter (ONSD) measurements for all 37 participants in the analytical sample across the six experimental conditions (Table S1); the complete generalized estimating equations (GEE) coefficient table that underlies the primary sensitivity analysis (Table S2); the absolute ONSD values by sex and condition (Table S3); a further sensitivity analysis excluding the participants who did not complete the full protocol (Table S4); and the correlations between the ONSD response and the concurrent systemic hemodynamic response (Table S5). All tables are referenced from the main manuscript.

2 NOTES

2.1 Software and reproducibility

All statistical analyses underlying Table S2 were carried out in Python 3.11 using the `statsmodels` library (`MixedLM` for the linear mixed-effects model and `GEE` with `Independence`-vs-`Exchangeable` covariance for the sensitivity analysis). The analysis scripts (`_gee_sensitivity.py` for Table S2 and `_round3_reviewer_analyses.py` for Tables S3–S5) and the input data underlying Table S1 are available from the corresponding author on reasonable request.

2.2 Estimator convergence

Both estimators converged without warnings. The random-intercept variance in the LMM was estimated at the boundary of the parameter space (singular fit), consistent with negligible between-subject variability in the proportional ONSD response after baseline referencing; the GEE approach, which does not rely on a between-subject variance component, gave estimates and inferences that were practically identical to the LMM, supporting the robustness of the primary analysis.

Table S1. Supplementary Table S1. Individual optic nerve sheath diameter measurements (mm) for the 37 participants of the analytical sample (18 female, 19 male) across the six experimental conditions (Baseline, LBNP10, LBNP20, LBNP30, LBNP40, Recovery). Missing values ("—") correspond to time points at which the participant did not complete the protocol due to presyncopal symptoms or at which a valid ultrasound measurement could not be obtained. Seven additional enrolled participants (IDs 1, 9, 11, 17, 35, 43, 44) were excluded from the analytical sample because no valid ONSD data were available at any time point and are not listed here. Sex codes: F, female; M, male.

ID	Sex	Baseline	LBNP10	LBNP20	LBNP30	LBNP40	Recovery
2	F	5.49	4.73	4.20	5.03	4.49	5.59
7	F	5.30	4.88	5.86	4.76	4.57	5.58
8	F	5.51	4.87	4.62	4.43	4.44	5.47
10	F	6.45	6.14	6.13	5.63	5.66	6.48
12	F	5.39	4.66	4.40	4.30	4.10	4.90
13	F	4.86	4.75	4.44	4.26	4.09	4.70
16	F	4.84	4.94	4.56	4.66	4.39	5.09
22	F	4.30	4.24	4.13	3.92	3.73	4.22
23	F	5.24	5.21	4.81	4.80	4.40	5.28
24	F	4.56	4.57	—	—	—	—
25	F	4.90	5.04	4.44	4.47	4.21	5.55
29	F	5.76	5.74	5.31	4.60	—	6.01
36	F	5.54	4.88	5.31	4.82	4.76	5.20
38	F	5.33	5.00	4.59	4.36	4.20	5.07
39	F	6.31	6.38	5.47	5.14	—	6.80
40	F	4.20	4.12	4.08	3.83	3.73	4.46
41	F	5.26	5.02	4.50	4.32	4.21	5.12
42	F	4.49	4.30	4.02	3.77	3.69	5.12
3	M	6.42	6.35	6.05	5.69	5.76	6.53
4	M	6.09	5.81	5.36	5.33	5.13	6.07
5	M	5.94	6.06	5.90	5.54	5.46	6.46
6	M	7.31	6.72	5.55	5.71	5.29	6.86
14	M	5.32	4.94	4.68	4.54	4.40	5.00
15	M	5.49	5.56	4.86	4.89	4.77	5.71
18	M	5.89	5.65	5.60	5.73	5.31	5.97
19	M	4.97	4.57	4.66	4.68	4.47	5.08
20	M	4.72	4.62	4.35	4.32	4.31	5.24
21	M	5.23	5.19	4.91	4.69	4.38	5.19
26	M	4.64	4.77	4.72	—	—	—
27	M	5.64	5.67	5.39	5.59	4.54	6.04
28	M	5.79	5.12	5.00	4.77	4.42	5.31
30	M	4.56	4.45	3.95	4.31	4.42	4.83
31	M	4.78	4.69	4.50	4.43	4.33	5.23
32	M	5.07	4.84	4.66	4.57	4.32	5.09
33	M	5.64	5.67	5.38	5.08	—	5.79
34	M	5.49	5.09	5.03	4.86	4.82	5.56
37	M	4.75	4.73	4.61	3.92	—	5.11

Table S2. Supplementary Table S2. Full generalized estimating equations (GEE) coefficient table for the sensitivity analysis described in the main manuscript. GEE were fitted with an exchangeable working correlation structure and cluster-robust (sandwich) standard errors on the participant level. Reference categories: State = Baseline, Sex = Female. Population-averaged coefficients are reported. For comparison, the corresponding linear mixed-effects model (LMM) estimates from the main analysis (Table 3 of the main manuscript) are given alongside; the two estimators agree to within ± 0.001 across all terms. SE, standard error; p, two-sided Wald p-value.

Parameter	LMM			GEE		
	β	SE	p	β	SE	p
State main effects (Female reference)						
State: LBNP10 vs. Baseline	-0.044	0.013	0.001	-0.044	0.012	< 0.001
State: LBNP20 vs. Baseline	-0.091	0.014	< 0.001	-0.091	0.018	< 0.001
State: LBNP30 vs. Baseline	-0.132	0.014	< 0.001	-0.132	0.012	< 0.001
State: LBNP40 vs. Baseline	-0.159	0.014	< 0.001	-0.160	0.009	< 0.001
State: Recovery vs. Baseline	$+0.018$	0.014	0.173	$+0.018$	0.015	0.217
Sex main effect (Baseline reference)						
Sex: Male vs. Female	~ 0	—	~ 1	~ 0	—	~ 1
State × Sex[Male] interactions						
LBNP10 × Sex[Male]	$+0.014$	0.019	0.452	$+0.014$	0.015	0.354
LBNP20 × Sex[Male]	$+0.012$	0.019	0.534	$+0.012$	0.022	0.592
LBNP30 × Sex[Male]	$+0.031$	0.019	0.108	$+0.031$	0.017	0.072
LBNP40 × Sex[Male]	$+0.025$	0.020	0.198	$+0.025$	0.017	0.142
Recovery × Sex[Male]	$+0.007$	0.019	0.733	$+0.007$	0.020	0.737

Table S3. Supplementary Table S3. Absolute optic nerve sheath diameter (ONSD, in mm; mean $\pm$ SD) at each experimental condition, separately for female and male participants of the analytical sample. The number of participants contributing a valid measurement (n) is given below each value; n decreases at the higher LBNP levels because some participants terminated the protocol early because of presyncopal symptoms. These absolute values correspond to the relative changes analyzed in the main manuscript.

Sex	Baseline	LBNP10	LBNP20	LBNP30	LBNP40	Recovery
Female	5.21 ± 0.62<br>$(n = 18)$	4.97 ± 0.60<br>$(n = 18)$	4.76 ± 0.63<br>$(n = 17)$	4.54 ± 0.48<br>$(n = 17)$	4.31 ± 0.49<br>$(n = 15)$	5.33 ± 0.66<br>$(n = 17)$
Male	5.46 ± 0.70<br>$(n = 19)$	5.29 ± 0.65<br>$(n = 19)$	5.01 ± 0.55<br>$(n = 19)$	4.92 ± 0.56<br>$(n = 18)$	4.76 ± 0.48<br>$(n = 16)$	5.62 ± 0.60<br>$(n = 18)$

Table S4. Supplementary Table S4. Sensitivity analysis excluding non-completers. Fixed-effect estimates from the linear mixed-effects model refitted after excluding the seven participants who terminated the LBNP protocol early because of presyncopal symptoms (31 participants remaining), compared with the primary analysis of the full analytical sample (37 participants; Table 3 of the main manuscript). Estimates (β) are relative changes from individual baseline; State coefficients are female-specific effects, and Male contrasts are State + State × Sex[Male]. p, two-sided Wald p-value. Every LBNP-induced reduction remains statistically significant and of similar magnitude in the reduced sample.

Group	State	Full ($n = 37$)		Excl. non-compl. ($n = 31$)	
		β	p	β	p
Female (State)	LBNP10	-0.044	0.001	-0.054	< 0.001
	LBNP20	-0.091	< 0.001	-0.090	< 0.001
	LBNP30	-0.132	< 0.001	-0.125	< 0.001
	LBNP40	-0.159	< 0.001	-0.161	< 0.001
	Recovery	$+0.018$	0.173	$+0.012$	0.399
Male (contrast)	LBNP10	-0.030	0.021	-0.038	0.007
	LBNP20	-0.079	< 0.001	-0.091	< 0.001
	LBNP30	-0.101	< 0.001	-0.099	< 0.001
	LBNP40	-0.134	< 0.001	-0.137	< 0.001
	Recovery	$+0.025$	0.058	$+0.020$	0.155

Table S5. Supplementary Table S5. Correlations between the relative change in ONSD and the relative changes in systemic hemodynamic variables (stroke volume, cardiac output, heart rate) across the four LBNP levels (LBNP10–LBNP40). Repeated-measures correlation (r_{rm}; Bakdash and Marusich, 2017) accounts for the within-participant structure of the data; ordinary (pooled) Pearson correlations are shown for comparison. All correlations were statistically significant (p < 0.001).

**ONSD vs.**	r_{rm}	p (r_{rm})	**Pearson** r	p **(Pearson)**
Stroke volume	$+0.64$	< 0.001	$+0.50$	< 0.001
Cardiac output	$+0.48$	< 0.001	$+0.29$	< 0.001
Heart rate	-0.53	< 0.001	-0.49	< 0.001

$N = 137$ paired observations from $k = 36$ participants.
